# Characterization and properties of hybrid foams from nanocellulose and kaolin-microfibrillated cellulose composite

**DOI:** 10.1038/s41598-020-73899-z

**Published:** 2020-10-15

**Authors:** Alina S. González-Ugarte, Islam Hafez, Mehdi Tajvidi

**Affiliations:** 1 Department of Chemical Engineering, University of Puerto Rico, Mayagüez, PR USA; 2 School of Forest Resources, University of Maine, Orono, ME 04469 USA; 3 Advanced Structures and Composites Center, University of Maine, Orono, ME 04469 USA

**Keywords:** Pollution remediation, Sustainability

## Abstract

Hybrid nanocellulose-based foams are a desirable class of low-density and porous materials for their potential in many applications. This study aims at characterizing and understanding the structure-properties relationship of four foam formulations prepared from combinations of cellulose nanofibrils (CNF), cellulose nanocrystals (CNC), and kaolin-microfibrillated cellulose composite. All the foams were crosslinked with a polyamide-epichlorohydrin crosslinker (Polycup) to impart stability under wet conditions without additional functionalization. Foams containing 25 wt% kaolin exhibited excellent shape recovery promoted by a higher load of crosslinker (5 wt%), and superior compressive properties. The addition of CNC at 33.3 wt% and 50 wt% did not seem to enhance the properties of the foam and also reduced the specific surface area. A preliminary comparative study between the four tested formulations was conducted to assess the feasibility of the foam as an adsorbent of methylene blue dye.

## Introduction

Nanocellulose-based foams are low-density and porous materials that have tremendous potentials in a myriad of applications^1^ such as environmental and biomedical applications. Cellulose is a homopolymer composed of β-d-glucopyranose units that are linked together by β-(1 → 4)-glycosidic bonds^2^. Cellulose nanofibrils (CNF) and cellulose nanocrystals (CNC) are two major types of cellulosic nanomaterials. CNFs are obtained through mechanical refining of wood and plant fibers^3^. CNCs are rod-like or whisker shaped particles remaining after acid hydrolysis of wood fiber, plant fiber, microcrystalline cellulose, microfibrillated cellulose, or nano-fibrillated cellulose. Both CNF and CNC are characterized by diameters in the nano-dimension and aspect ratio ranging between 5 and 50^4^.

Nanocellulose-based foams can be produced through a number of techniques most commonly freeze-drying and supercritical CO_2_ drying. Freeze-drying is a well-established and simple technique capable of producing low-density foams from nanocellulose^5^. This technique relies on the removal of water via sublimation which occurs when the solvent (water) temperature is below the triple point (the temperature and pressure at which solid, liquid, and vapor phases of a substance coexist in equilibrium).

Hybrid nanocellulose-based foams and aerogels have gained a lot of interest because of their potential to introduce multifunctionality into the foamed structure^6–8^. Many studies have investigated the use of clay with nanocellulose in cellulose-reinforced clay aerogels and foams^9–11^. Long et al. successfully prepared cellulose and montmorillonite aerogels via a sol–gel process, solvent exchange, and freeze-dying. The study reported that montmorillonite adhered to the cellulose surface through hydrogen bonding. Ahmadzadeh et al. also reported improvements in compressive strength and Young’s modulus as a result of the addition of montmorillonite nanoclay. This hybrid foam was intended to be used for packing food products as an alternative to commercial synthetic foams. Hybrid cellulose nanofibrils and sepiolite clay have shown superior flame retardancy and thermal insulation resulting from the addition of clay^12^.

Kaolin is a low-cost and abundant clay that can be well-dispersed in CNF suspensions^13^. The incorporation of kaolin into cellulosic-based foamed structures to produce environmentally friendly materials suitable for a number of applications remains an unexplored area of research. Kaolin has a chemical formula of Al_2_Si_2_(OH)_4_ and is composed of stacked pairs of tetrahedral silicate (Si_2_O_5_) and octahedral alumina sheets (Al_2_(OH)_4_)^14–16^. The 1:1 pairs of layers are held firmly by hydrogen bonding, which explains why kaolin clays do not swell. Cellulose and kaolin hybrid materials were mainly studied in non-foam products such as printed electronics and paper production. The addition of kaolin lowered the cost and improved the optical properties of paper^17^. It was also used with bacterial cellulose for the production of wound healing materials^18^. The incorporation of kaolin in nanocellulose-based foams could add economic viability to bio-based products and pave the way to further developments and advancements.

Cellulosic nanomaterials have been researched extensively for their application in water purification owing to their outstanding properties^19^. Most of the previous attempts that targeted methylene blue dye have not been successful without functionalization steps. These functionalization steps usually add to the complexity and cost of the final product. Martins et al.^20^ reported a maximal removal capacity of up to 86% for their hydroxypropyl methylcellulose (0.12 ± 0.001 g aerogel). Surface-functionalization of TEMPO aerogels achieved by coating with eumelanin thin films has resulted in a methylene blue adsorption of up to 50% (58 mg average and adsorbent in a 2 mg/L methylene blue)^21^. Wu et al.^22^ combined freeze-drying and supercritical drying to prepare crosslinked cellulose nanofibril aerogels for methylene blue removal with high efficiency. It is apparent that, to prepare highly-efficient aerogels-based adsorbent from cellulosic nanomaterials, several functionalization reactions or energy-intensive processes are needed. In addition to nanocellulose, kaolin has the ability to decontaminate wastewater from organic pollutants^23^. Color in water could be removed by scavenging via clays in soil. There have been many recent studies highlighting the proof of concept of using unmodified and modified kaolin as adsorbents for methylene blue dye^14,15,24,25^.

To the best of our knowledge, combining nanocellulosic materials and kaolin clay into a foamed structure remains unexplored. Most of the previous attempts have focused on using nanocellulose with montmorillonite (usually as a reinforcing agent)^7,26^. We believe that combining kaolin and nanocellulose into foamed structures and understanding their properties will expand the application span of bio-based foams and pave the way for further development of new materials. Recently, a composite type of micro and nano-fibrillated cellulose containing various types of minerals has been commercialized, which has the potential to accelerate global efforts to find eco-friendly alternative materials for many applications^27^.

The aim of this study is to characterize and understand the correlation between the structure of nanocellulosic materials/kaolin foams prepared via freeze-drying and their properties. The morphology of the foam, compressive properties, shape recovery, crosslinking reaction with an epichlorohydrin-based crosslinker, and thermal stability were studied. In addition, a preliminary comparative study has been conducted to assess the adsorption capability of methylene blue onto the foam.

## Materials and methods

### Materials

Cellulose nanofibrils (CNF) of 3 wt% solid content was obtained from the Process Development Center (PDC) of the University of Maine and it was used as received. The CNF was produced from bleached softwood Kraft pulp through a mechanical refining process. Cellulose nanocrystals (CNC) of 12.1 wt% solid content and 0.95% sulfur on dry sodium form CNC was also obtained from the PDC and used as received. Kaolin-based microfibrillated cellulose (50:50 mix) press cake (34 wt% solid content) was obtained from FiberLean Technologies, UK. This press cake was further diluted to a solid content of 3.6 wt% prior to the preparation of foams. Polycup 5150 crosslinker (26 wt% solid content) was purchased from Solenis (Wilmington, DE, USA). Methylene blue trihydrate and silicone oil were purchased from Thermo Fischer Scientific (Waltham, MA, USA).

### Preparation of the foams

Different foam formulations were prepared by mixing nanocellulose and kaolin-based micro-fibrillated cellulose (FLK) according to Table 1. This FLK is used as the source of kaolin clay and it contains approximately 50 dry wt% of kaolin. The mixing was conducted at room temperature in 150-mL beaker using a spatula for approximately five minutes until a homogenous mixture was obtained. Polycup was subsequently added at 1 wt% or 5 wt% loading levels based on the total dry mass of the foam. The mixture was poured into a cylindrical plastic mold and subsequently freeze-dried in a Harvest Right freeze dryer (North Salt Lake, Utah, USA). The freeze-drying process was performed at temperature cycles of − 34.4, − 6.7, 4.4, 15.6, and 32.2 °C for 5, 10, 8, 3, 3 h, respectively. After drying, the crosslinking was induced by heating the foams in a vacuum (25 inHg = 86 kPa) oven at 105 ± 2 °C for three hours.

**Table 1:** Summary of the foam formulations.

Sample name	CNF		CNC		FLK		Crosslinker	
	CNF (%)	CNF dry mass (g)	CNC (%)	CNC dry mass (g)	FLK (%)	FLK dry mass (g)	Crosslinker wt%	Crosslinker dry mass (g)
Neat CNF (0%)^a^	100	0.13	0	0	0	0	0	0
Neat CNF (5%)	100	0.13	0	0	0	0	5	0.0065
CNF:CNC (5%)	50	0.065	50	0.065	0	0	5	0.0065
CNF:FLK (5%)	50	0.065	0	0	50	0.065	5	0.0065
CNF:CNC:FLK (5%)	33.3	0.043	33.3	0.043	33.3	0.043	5	0.0065
Neat CNF (1%)	100	0.13	0	0	0	0	1	0.0013
CNF:CNC (1%)	50	0.065	50	0.065	0	0	1	0.0013
CNF:FLK (1%)	50	0.065	0	0	50	0.065	1	0.0013
CNF:CNC:FLK (1%)	33.3	0.043	33.3	0.043	33.3	0.043	1	0.0013

^a^Values within parentheses in the sample name column correspond to weight percent of the crosslinker.

### Characterization

The morphology of the foams was examined by scanning electron microscopy (SEM). A Zeiss NVision 40 microscope was used to assess samples obtained from a longitudinal slice resulting from a vertical cut of the foam. The samples were coated with gold/palladium to enable the imaging of samples. All images were taken at an accelerating voltage of 3 kV.

Compressive properties were evaluated using an Instron 5942 (Norwood, MA, USA) equipped with a 500 N load cell. The tests were conducted according to ASTM C165 at a test speed of 0.0635 mm/min. Prior to testing, the samples were conditioned in a conditioning chamber at a relative humidity of 53 ± 2% and a temperature of 23 ± 2 °C for 24 h.

Infra-red spectroscopy was conducted using a Perkin Elmer UATR 2 (Waltham, MA, USA) equipment to assess the different types of chemical bonds resulting from the crosslinking of nanocellulose. The samples were tested within a range of 500–4500 cm^−1^ and all spectra were normalized with respect to wavenumber 1055 cm^−1^ which corresponds to the stretching vibration of the cellulose backbone (not altered by the crosslinking reaction).

Water absorption capacity was determined by measuring the mass of foams before submerging in water ($w_{i}$) and the mass after submerging in 50 mL of distilled water ($w_{f}$) according to Eq. (1).

1 $${\text{Water absorption capacity }}\left( {{\text{g g}}^{{ - {1}}} } \right) \, = \frac{{{\text{W}}_{{\text{f}}} - {\text{W}}_{{\text{i}}} }}{{{\text{W}}_{{\text{i}}} }}.$$

To calculate the density $\left( \rho \right)$ of foams, the volume (measured by a digital caliper) and mass of foams were measured. The density was calculated according to Eq. (2), where (*m*) and (*v*) are mass and volume of foams, respectively.

2 $$\rho _{foam} = \frac{m}{v}.$$

The specific surface area was measured using a Micromeritics ASAP 2020 analyzer through the BET (Brunauer–Emmett–Teller) N_2_ adsorption method. The samples were degassed at 75 °C for five hours under vacuum.

The assessment of the shape recovery functionality was conducted by compressing the foams using a Dake manual hydraulic pump at a pressure of 1.2 kPa. The formed thin sheet of foam (0.5–0.7 mm) was subsequently soaked in 50 mL of distilled water for 10 s.

The foam’s height before compression (*h*_*i*_) and height after soaking in water (*h*_*f*_) were recorded. The shape recovery (%) was calculated according to Eq. (3).

3 $${\text{Shape}}\,{\text{recovery }}\left( \% \right) = \frac{{{\text{h}}_{{\text{f}}} }}{{{\text{h}}_{{\text{i}}} }}{ } \times {100}{\text{.}}$$

The mass loss after compression and soaking was determined gravimetrically according to Eq. (4). The foam sample was dried at 75 °C for 5 h or until a constant mass was reached and the dried mass was determined (*w*_*i*_). The foam was compressed, soaked in water and then redried at the same temperature until a constant mass was reached (*w*_*f*_).

4 $$Mass\,loss \left( \% \right) = \frac{{w_{i} - w_{f} }}{{w_{i} }} \times 100.$$

The porosity of foams was determined by the silicone oil void measurement technique modified from Hossen et al.^28^. In this method, the pores volume was obtained by calculating the volume silicone oil penetrated inside the pores of the foam. First, the volume of foam (cylindrical shape) was determined using a digital caliper. The foams were degassed at 70 °C for 2 h in a vacuum (25 inHg = 86 kPa) oven and the mass of degassed foams (*w*_*i*_) was recorded. The foams were subsequently submerged in silicone oil for 2 h. The used silicone oil had a viscosity of 0.0432–0.055 Pa.s at 25 °C and a density (*ρ*_so_) of 0.96 g/mL at the same temperature. The excess oil was removed from the surface and the mass of foams containing silicone oil (*w*_*f*_) was determined. The porosity (%) was calculated according to Eq. (5).

5 $$Porosity \left( \% \right) = \frac{{\left( {W_{f} - W_{i} } \right)/\rho_{so} }}{Total\,foam\,volume} \times 100.$$

The thermal stability of foams was evaluated via thermogravimetric analysis using a TGA Q500 (TA Instruments, New Castle, DE, USA). The test was conducted from room temperature (~ 25 °C) to 600 °C at a heating rate of 10 °C/min under a nitrogen atmosphere at a flow rate of 40 mL/min.

### Adsorption experiment

A methylene blue stock solution of 100 mg/L was prepared and followed by the preparation of the following concentrations: 1, 3, 5, 7, and 10 mg/L. The prepared concentrations were used to generate a calibration curve. The absorbance of methylene blue of each concentration at $\lambda_{max}$ = 668 nm was measured by a Beckman Coulter DU-7500 UV–Visible Scanning Spectrophotometer (Brea, CA, USA).

To determine the removal efficiency, foams were submerged in a 5 mg/L MB solution contained in a 50 mL tube. The tubes were gently agitated for 24 h using a VWR Scientific Products rocking platform Model 100 (Radnor, PA, USA). After 24 h, the absorbance of the unknown concentration of methylene blue was determined at the same $\lambda_{max}$ (668 nm). The unknown concentration was calculated from the linear equation of the calibration curve and the removal efficiency was determined using Eq. (6).

6 $${\text{Removal}}\,{\text{efficiency }}\left( \% \right) \, = \frac{{{\text{(C}}_{{\text{o}}} - {\text{C}}_{{\text{e}}} {)}}}{{{\text{C}}_{{\text{o}}} }} \times 100,$$

where (*C*_*o*_) is the initial concentration and (*Ce*) is the concentration after 24 h.

One-way analysis of variance (ANOVA) and independent T-test were used to determine the statistical difference between the means of the independent variables. Duncan’s Multiple Range Test (DMRT) was conducted as a post hoc test to evaluate the specific differences between pairs of means. All analyses were performed using IBM SPSS Statistics 25 software at a 95% confidence level.

## Results and discussions

### Internal structure and mechanical properties

**Figure 1:**
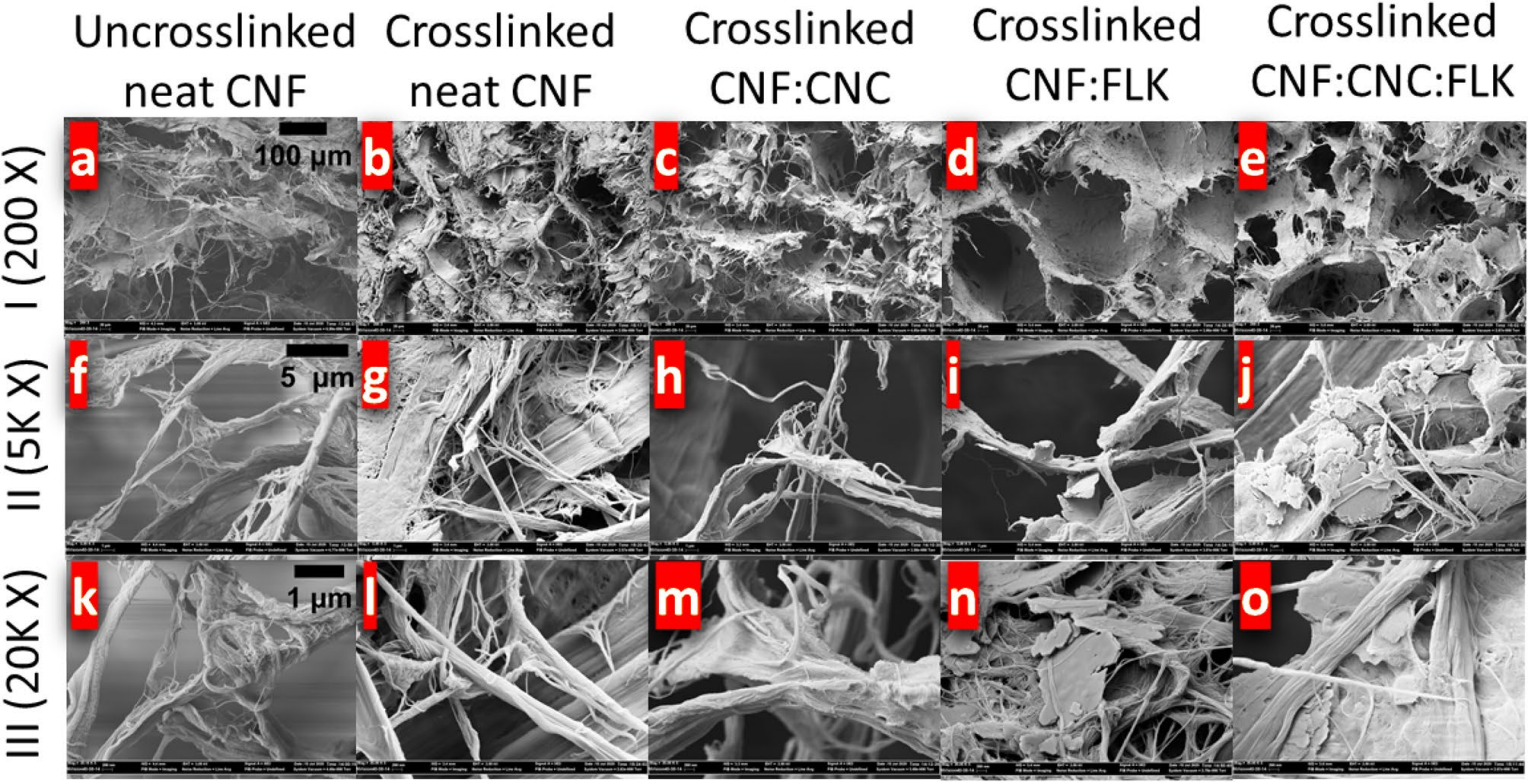
SEM images of nanocellulose-based foams at different magnifications.

**Figure 2:**
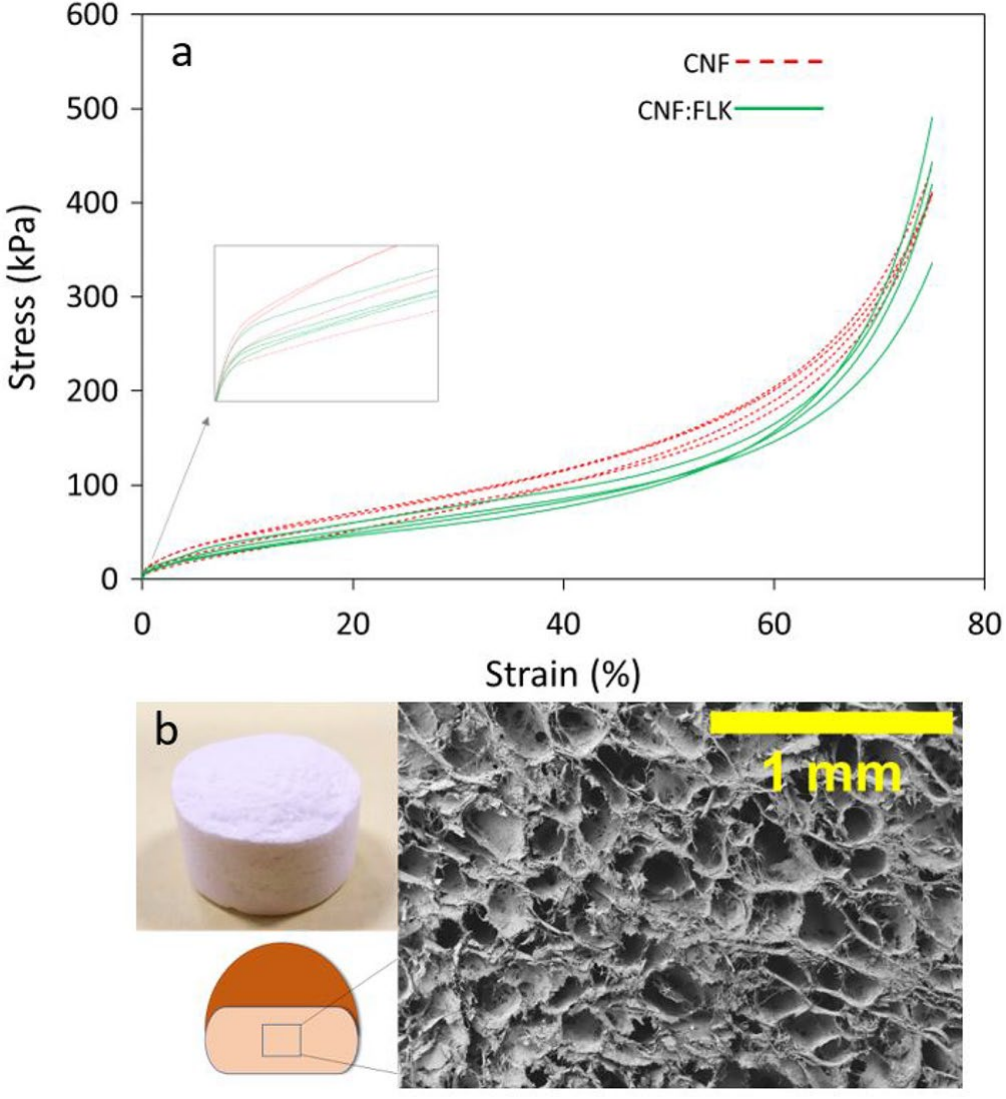
Compressive stress–strain curves of CNF and CNF:FLK samples (**a**), and a photograph taken at the Laboratory of Renewable Nanomaterials at the University of Maine and SEM images of CNF:FLK (**b**).

The porosity of the prepared foams ranged between 90 and 96% as determined by the silicone oil void measurement technique. To examine the internal structure of the foams, scanning electron microscopy (SEM) was used. The evaluated samples were neat CNF without and with a crosslinker, CNF:CNC, CNF:FLK, and CNF:CNC:FLK, all at a 1 wt% crosslinker loading level (Fig. 1). One can observe the free ‘hanging’ fibrils in the uncrosslinked sample of the neat CNF foam (Fig. 1a). By contrast, these free fibrils were absent in the crosslinked sample and the fibrils were interconnected together as a result of crosslinking (Fig. 1b). This observation of interconnected fibrils in neat CNF was also consistent with the other crosslinked samples. A closer observation shows a distinctive pattern in the CNF:FLK sample (Figs. 1d, 2b), where the shape of the foam cells was more regular than the other foam samples. Higher-magnification images showed the dimensions of the individual fibrils ranging from 50 to 500 nm in diameter. The kaolin particles have dimensions ranging between 500 nm and 2 µm in length and width and they appear to be surrounded and held in place by the individual fibrils (Fig. 1n,j).

In order to gain insight into the relationship between the structure and mechanical properties of the foam, a compressive testing was conducted. The compressive testing focused on the samples with and without kaolin (neat CNF vs CNF:FLK). Typical compressive stress–strain curves (Fig. 2a) show a linear elastic region at low strains. The middle region of the curve is a gradual plastic deformation (1–50%) followed by a densification region. The compressive strengths were determined at 10%, 25%, and 70% strain values (Table 2). The stress–strain curves of the two samples appeared similar except in the region that preceded the densification. An independent T-test revealed that there was no significant difference between the compressive strength values of CNF and CNF:FLK foams at 10% (p-value; 0.279) and 70% (p-value; 0.094) strains, respectively, at 95% confidence level. At 25% strain, however, the CNF sample exhibited a significantly higher compressive strength than CNF:FLK. This is likely due to the decrease in the fibrils content and hence a reduction in the fibril-fibril hydrogen bonding which allowed easier compression in the plastic region. The compressive moduli of the two samples were not significantly different. The compressive moduli were calculated as 4.1 and 3.2 MPa for CNF (density; 0.037 g/cm^3^) and CNF:FLK (density; 0.040 g/cm^3^) samples, respectively, from the linear elastic region. This is an unanticipated, yet interesting, finding since it was hypothesized that the samples containing kaolin clay would exhibit less stiffness as opposed to the neat CNF sample. The reported compressive modulus for CNF foam of a density of 0.063 g/cm^3^ was around 1.76 MPa^29^ which is less than half of the compressive modulus of our foam. The foam in the reported study was also produced by freeze-drying, however, there were differences in the freezing method, compression test speed (1.4 mm/min), and the source of CNF. The CNF used in the published study had larger diameter than our CNF (30 µm vs 50–500 nm). Our foams were dried at a low rate using a built-in chiller in the freeze-dryer whereas the reported foam was dried rapidly using liquid nitrogen. In the CNF:FLK sample, it seems possible that the enhanced compressive properties was due to the distinctive pattern of this sample (Figs. 1d, 2b) which presumably gives an additional mechanical support to the foam. Previous studies have focused on using CNF at low loading levels as reinforcing agents in clay-based foams^7,26^. These studies demonstrated the improvement in compressive properties with the increase in CNF content.

**Table 2:** Summary of the compressive property results.

	Compressive modulus (MPa)	Stress_10_ (kPa)	Stress_25_ (kPa)	Stress_70_ (kPa)
CNF	4.1 (1)^a^	41 (9)^a^	73 (8)^a^	302 (12)^a^
CNF:FLK	3.2 (0.4)^a^	36 (5)^a^	60 (7)^b^	272 (27)^a^

Similar letters within each column denote no significant difference at 95% confidence level.

^a^Values within parentheses represent the standard deviation.

### Fourier transform infra-red (FTIR) analysis

**Figure 3:**
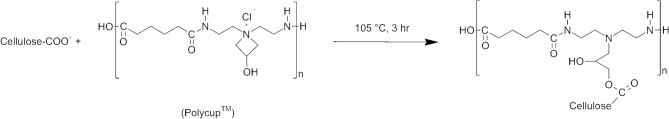
Proposed reaction mechanism between cellulose and Polycup.

Heat curing of the foams initiated a reaction between the cellulose and polyamide-epichlorohydrin crosslinker (Polycup 5150). Polycup contains four-membered 3-hydroxy-azetidinium groups on most of the repeating units^30^. The proposed crosslinking mechanism is thus through the interaction between the azetidinium group and carboxyl groups of nanocellulose, leading to the formation of ester linkages (Fig. 3).

**Figure 4:**
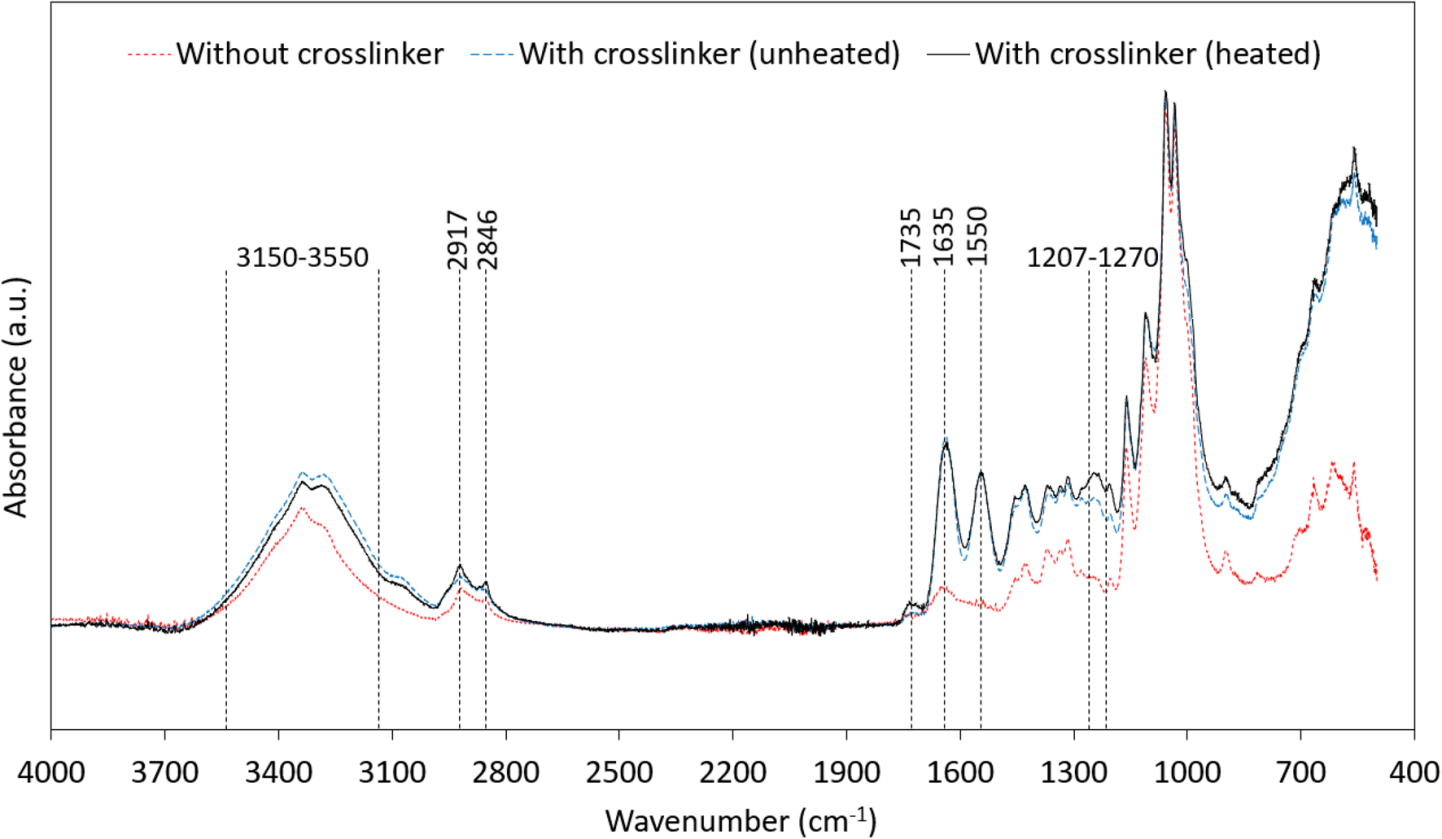
FTIR spectra of neat CNF foam without crosslinker, with crosslinker (heated; i.e. crosslinked), with crosslinker (unheated; i.e. crosslinking has not been initiated).

All the FTIR spectra (Fig. 4) were normalized by the band 1055 cm^−1^ which corresponds to the stretching vibration of the cellulose backbone and is not altered by the crosslinking reaction^31^. The broad peak between 3150 and 3550 cm^−1^ was attributed to the OH stretching of CNF^32^. This broad peak had a higher intensity in the samples with crosslinker (unheated and heated) compared to the sample without a crosslinker. This increase in intensity was attributed to the N–H stretching^33^ which overlaps with the hydroxyl groups of CNF in the same region. In addition, an increasing trend in the bands 2917 and 2846 cm^−1^ was observed in the following order: uncrosslinked CNF < with crosslinker (unheated) < with crosslinker (heated). These two bands correspond to the aliphatic C–H stretching^34^. The increase in this peak in the unheated sample (compared to CNF without crosslinker sample) is believed to result from the presence of the crosslinker which contains multiple aliphatic C–H bonds on its backbone. The additional increase in the heated sample is presumably due to the increase in aliphatic C–H attributable to the ring-opening of azetidinium group upon heating.

The intensity of C=O stretching vibration of ester bonds (1735 cm^−1^)^30^ in the heated sample was higher compared to the other two samples verifying the formation of ester bonds between CNF and Polycup. The bands 1635 and 1550 cm^−1^ corresponded to amind I and amid II in both heated and unheated samples^30^. The broad peak that arises between 1208–1270 cm^−1^ is attributed to C–O stretching vibration of ester bonds^30,35^. This peak had the highest intensity for the heated samples, whereas the lowest intensity was observed for the uncrosslinked sample, which further verifies the formation of the ester bonds.

### Water absorption capacity, BET surface area, and shape recovery

Table 3 summarizes the water absorption capacity results for foams crosslinked at 1 wt% and 5 wt% loading levels. The results of both 1 wt% and 5 wt% crosslinker were comparable which indicates that the extent of crosslinking (i.e. the extent of ester linkages) did not affect the capacity of water held by the foams. What is interesting in this result is the change of absorption capacity among the different samples at the same crosslinker loading level. Overall, the absorption capacities of the foams ranged from 13 to 20 times their own weight. Foams prepared from neat CNF exhibited a water absorption capacity of 19 g/g (for the two crosslinker loading levels). The strong affinity of CNF towards water is a result of the abundance of hydroxyl groups^36^.

**Table 3:** Water absorption capacity, densities, and BET surface area of the foams.

Sample	Water absorption capacity (g/g)		Density (g/cm^3^)		BET surface area (m^2^/g)
	1 wt% crosslinker	5 wt% crosslinker	1 wt% crosslinker	5 wt% crosslinker	1 wt% crosslinker
Neat CNF	18.9 (1.5)^a^	19.0 (0.8)	0.037 (0.002)	0.047 (0.007)	9.4
CNF:CNC	13.0 (1.3)	13.2 (0.4)	0.061 (0.002)	0.075 (0.003)	2.6
CNF:FLK	19.2 (2.0)	20.0 (1.5)	0.040 (0.001)	0.042 (0.002)	7.1
CNF:CNC:FLK	14.4 (2.8)	14.1 (1.1)	0.053 (0.001)	0.056 (0.002)	2.4

^a^Values within parentheses represent the standard deviation.

CNF:FLK had an almost similar absorption capacity to neat CNF. Despite the presence of kaolin clay and a lower amount of CNF, this sample still exhibited comparable absorption capacity to neat CNF foam. This could be explained by the strong affinity of kaolin to bind water^37^. The affinity of kaolin to water depends on the type of layer in its structure (silicate vs. alumina) and the location at each layer (edge vs. surface). Typically, the two edges have a strong affinity to water whereas the surface of the layers has a weaker affinity^37^.

The lowest absorption capacities were 13 and 14 g/g for CNF:CNC and CNF:CNC:FLK samples, respectively. It appears that the decrease in the absorption capacity has resulted from the presence of CNC. It has been demonstrated that CNF has a higher equilibrium moisture content than CNC at a 95% relative humidity^38^ which is attributed to the presence of amorphous domains of CNF as opposed to CNC, hence a decreased absorption capacity for the CNC-containing samples.

The apparent volumetric mass density of foams ranged from 0.035 to 0.058 g/cm^3^ (Table 3). The uncrosslinked neat CNF sample had the lowest density of 0.035 g/cm^3^ (not shown in the table). As a general trend, the density increased with the increase of the extent of crosslinking (1 wt% vs. 5 wt%). Foams prepared at 1 wt% crosslinker loading level exhibited an average density of 0.048 g/cm^3^ whereas the average density of foams prepared at 5 wt% crosslinker was 0.058 g/cm^3^.

Furthermore, a correlation between water absorption capacity and density of foams was discerned. It was found that foams with lower densities exhibited higher water absorption capacities and vice versa. This correlation between density and water absorption was also observed in previous studies^39^. Our neat CNF foam had a lower water absorption capacity than the CNF aerogel prepared by Zhang et al.^31^ (19 g/g vs. 96 g/g). This is attributed to the variation in the starting solid content (3 wt% vs. 1 wt%) where a lower solid content leads to a higher water absorption capacity resulting from the decrease in density.

**Figure 5:**
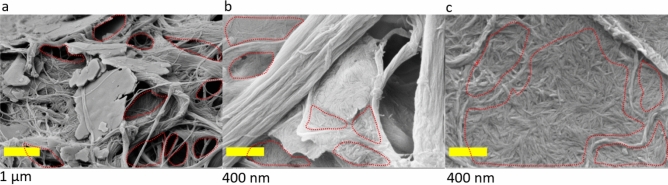
Open and closed voids in the presence and absence of CNC for CNF:FLK (**a**), CNF:CNC:FLK (**b**), and CNF:CNC (**c**) samples.

The BET specific surface area of the neat CNF foam is in agreement with the reported values of CNF foams obtained by freeze-drying, which demonstrates the macroporous nature of the foam^1,40^. The CNF:FLK sample had a specific surface area of 7.1 m^2^/g. The foams containing CNC exhibited a much lower specific surface area (2.4 and 2.6 m^2^/g). This result may be explained by the fact that CNCs fill the fibril-fibril and/or fibril-kaolin voids through non-porous and continous networks (Fig. 5) goverened by hydrogen bonding.

**Figure 6:**
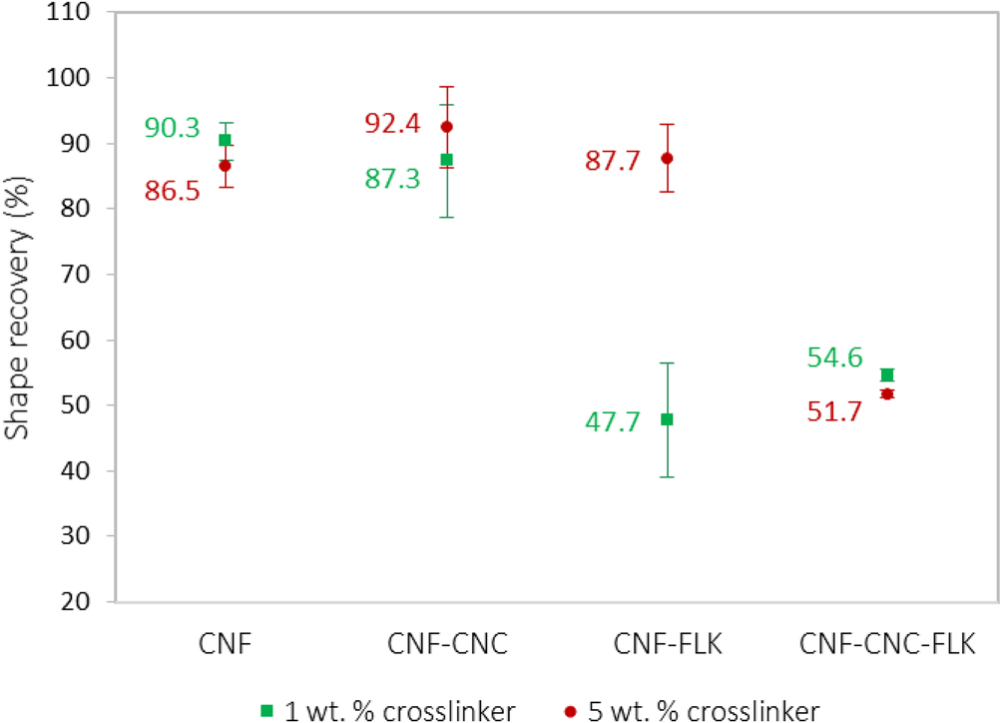
Shape recovery of foams as a function of formulation and amount of crosslinker.

Interestingly, the prepared foams exhibited shape fixation and shape recovery functionalities (Fig. 6). When the foams were subjected to a compression force (1.2 kPa), a thin sheet was formed (0.5–0.7 mm). These compressed foams did not recover their shape when placed in ambient conditions. Wet foams also maintained their shape after compression. When the compressed dry foams was placed in water, they recovered a substantial portion of their initial shape.

Foams prepared from neat CNF recovered 90% and 87% of their shape at 1 wt% and 5 wt% crosslinker loading levels, respectively. Foams prepared from CNF:CNC had comparable (to neat CNF) shape recovery results of 87% and 92% at the two crosslinker loading levels. The CNF:CNC:FLK sample exhibited a considerably poorer shape recovery of 55% and 52% at the same crosslinker levels, respectively. For those three samples, the shape recovery results at the two crosslinker levels were comparable. The most striking result to emerge from the data is the shape recovery of CNF:FLK which behaved differently between the two crosslinker levels. At 1 wt% crosslinker, CNF:FLK sample recovered only 48% of its shape, whereas at 5 wt% crosslinker, the foam recovered 88% of its initial shape.

Kim et al.^41^ attributed the shape fixation observed in compressed dry CNF aerogels to the bending of amorphous regions resulted from their low elastic modulus. Zhang et al.^31^ observed different deformation behavior between dry and wet aerogels prepared from cellulose nano-/microfibrils without additives. The dry aerogels, when subjected to a compression force, recovered about 20% of their shape in two minutes whereas wet aerogels exhibited a shape fixation property. The authors attributed the shape fixation observed ‘only’ in wet aerogels to the high surface tension of water which creates a large capillary force holding the fibrils together^42^. Upon wetting, more water diffuses into the pores^43^ followed by partial swelling of amorphous cellulose and expansion of fibrils. The authors also highlighted the importance of solubility parameters and the polarity of the solvent used to stimulate the aerogels^44^.

In our foams, the presence of kaolin and different types of nanocellulose rendered this process more complex, and thus further explanation was needed. It is presumed that the shape recovery in this study is attributed to three factors: (1) the fraction of CNF, (2) the degree of crosslinking, and (3) the type of coexisting entities. The favorable shape recovery of neat CNF foam (87–90%) is partly due to the presence of an adequate network of fibrils that enables sufficient swelling and expansion once placed in water. The crosslinking also plays a critical role in the shape recovery phenomenon. When uncrosslinked foams were placed in water, no shape recovery was noticed. The extent of crosslinking (1 wt% vs. 5 wt%) did not seem to significantly affect the recovery of the initial shape of this sample.

In CNF:CNC sample, it is apparent that CNC also plays a critical role in the shape recovery of foams despite the low portion of CNF. The modulus of elasticity of the crystalline domains of cellulose was estimated between 130 and 250 GPa^45^. This high modulus of elasticity is likely to contribute to the elastic recovery of strain^46^ when soaked in water. In other words, this mechanism compensates for the lower amount of CNF in this foam sample.

When replacing CNC by FLK (i.e. in CNF:FLK sample; containing a total of 25 wt% kaolin), the mechanism differs and a discrepancy between the two crosslinker levels was observed. In such a case, a higher extent of crosslinking appears to be necessary to crosslink the relatively lower portion of CNF. Stated differently, as the portion of CNF decreases, a crosslinker loading level of 5 wt% is needed. More data points (between 1 wt% and 5 wt% crosslinker), however, will need to be tested for this sample in order to determine the shape recovery trend. This assumption is presumably not valid beyond a certain CNF threshold as supported by the result of CNF:CNC:FLK sample where the CNF portion was 33 wt% and a higher level of crosslinker was not effective in inducing a shape recovery functionality to this sample. Overall, these findings indicate that the shape recovery property in the studied foams was a result of interaction between the fraction of CNF, type and load of the coexisting entity, and degree of crosslinking. Also, all the crosslinked foams at the two crosslinker loading levels exhibited stability upon wetting unlike the uncrosslinked sample which disintegrated once soaked in water. To eliminate the possibility of wetting the kaolin and hence causing a mass loss after compression and soaking of the foams, the amount of mass loss was determined gravimetrically. It was found that in the four tested samples the mass loss was only 1–2% which strongly suggests that no significant amount of kaolin has been removed during soaking that could lead to the poor shape recovery of the CNF:FLK sample at the low crosslinker level. It should be noted that the FiberLean material used in this study (the kaolin/microfibrillated cellulose composite) is produced by co-gringing wood pulp and kaolin and therefore a high interaction between kaolin and fibrilated cellulose is expected.

### Methylene blue dye removal efficiency

**Figure 7:**
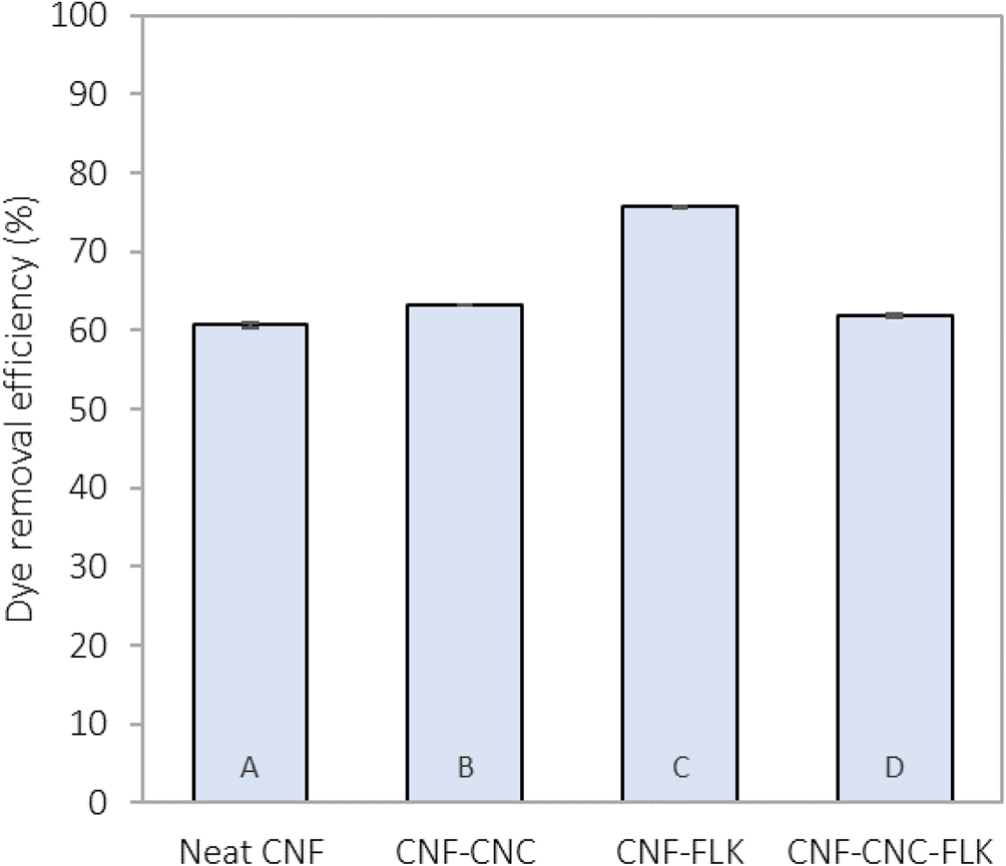
Dye removal efficiency of foams prepared with 1 wt% crosslinker after 24 h (initial dye concentration: 5 mg/L). Columns with common letters are not statistically different at a 95% confidence level.

The removal efficiency of methylene blue by the prepared foams is summarized in Fig. 7. Initially, the two crosslinker loading levels (1 wt% and 5 wt%) were tested for their removal efficiencies towards methylene blue dye. The foams prepared from the two crosslinker loading levels showed considerable stability when immersed in water for 24 h under continuous agitation. In this experiment, it was assumed that the system reaches an equilibrium at 24 h^47^. The uncrosslinked sample (from neat CNF), disintegrated once soaked in water and agitated. It was also found that all the foams formulations prepared with 5 wt% crosslinker loading level did not show any adsorption capability towards the dye; thus, the results are not shown in Fig. 7. This is probably due to the increased loading of crosslinker which results in the blockage of adsorption sites on the cellulosic entities.

The removal efficiency of the four formulations prepared with 1 wt% crosslinker loading level ranged from 61 to 76%. The highest removal efficiency (76%) was achieved by the foams prepared from CNF:FLK. The lowest removal efficiency (61%) was obtained by the neat CNF foam. The relatively higher efficiency of the CNF:FLK could be attributed to the presence of a larger amount of kaolin. Kaolin has been found efficient in the removal of contaminants especially methylene blue dye^48,49^. It is commonly known that the electrostatic attraction and hydrogen bonding between methylene blue and cellulose are the primary mechanisms that govern the adsorption of methylene blue onto the foam^50^. The chemical structure of kaolin contains hydroxyl groups that form hydrogen bonding with the amine groups from the dye^51^. The electrostatic attraction, however, depends on the pH of the solution^52^. The number of negative charges of kaolin increases with the increase of pH hence the enhancement of adsorption of the cationic dye^14^. The maximum adsorption of methylene blue onto kaolin occurred in a pH range of 6–10^53^. In this study, the experiments were conducted under neutral conditions (pH = 7). Comparing to other studies that used nanocellulose in the removal of methylene blue, Mohamed et al.^47^ used a cellulose nanocrystal membrane to adsorb this dye. The membrane resulted in a removal efficiency of 85% after 24 h for an initial concentration of 5 mg/L. Martins et al.^20^ reported a maximal removal capacity of up to 86% for their hydroxypropyl methylcellulose (0.12 ± 0.001 g aerogel). Surface-functionalization of TEMPO aerogels achieved by coating with eumelanin thin films resulted in a methylene blue adsorption of up to 50% (58 mg average and adsorbent in a 2 mg/L methylene blue)^21^. This shows that our foam is comparable to the reported aerogels even without the need to additional functionalization and in a one-step process.

### Thermal stability

**Figure 8:**
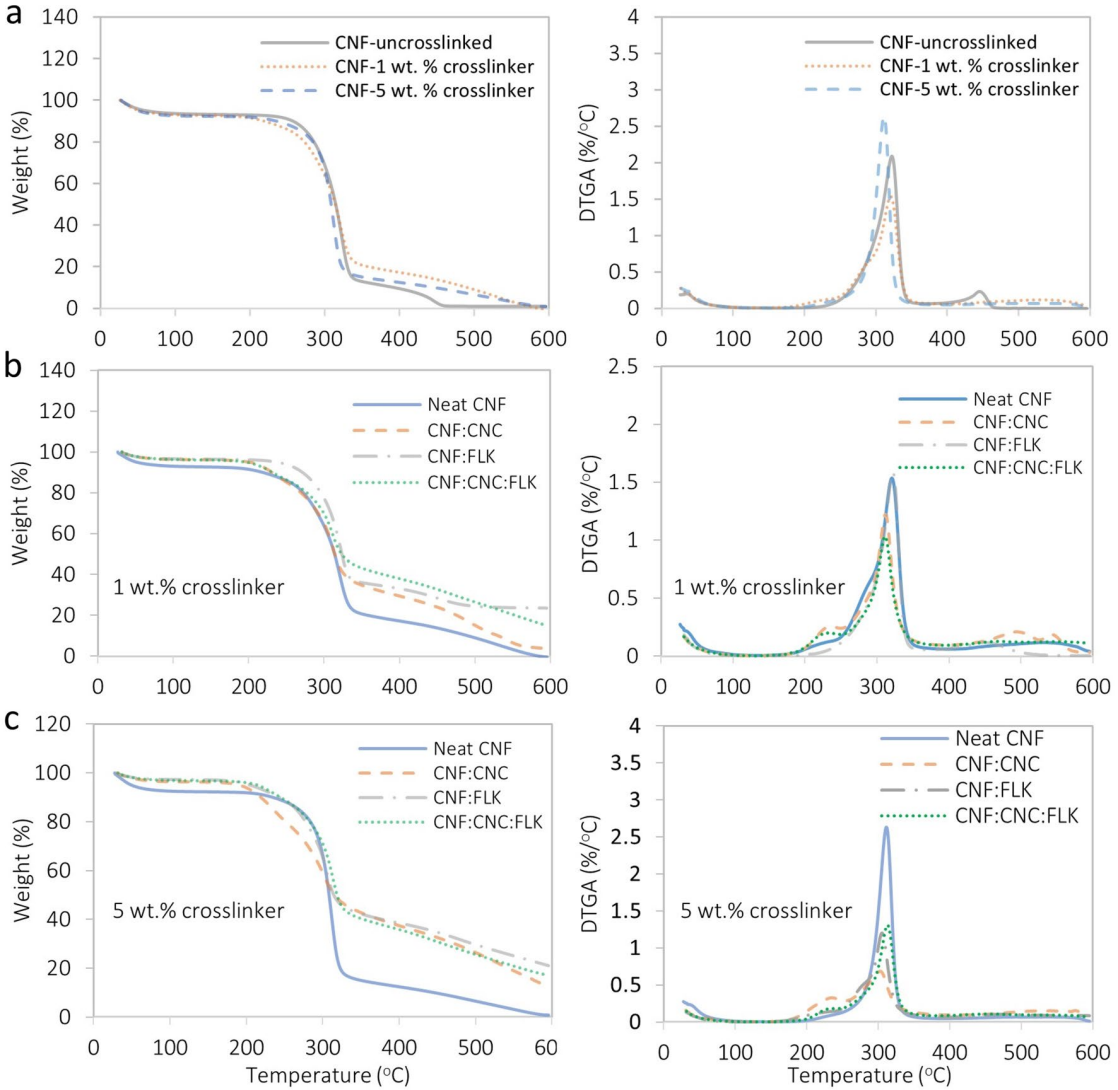
TGA (left) and DTGA (right) curves of uncrosslinked neat CNF, crosslinked CNF-1 wt%, and crosslinked CNF-5 wt% (**a**), foams from different combinations at 1 wt% crosslinker (**b**), and foams from different combinations at 5 wt% crosslinker (**c**).

The thermal stability of foams was studied by TGA and the results are shown in Fig. 8. On average, the initial weight loss occurring between room temperature and 150 °C was attributed to the loss of moisture from the foam. The weight loss between 275 and 450 °C was attributed to the cleavage of the glycosidic bond between the glucose units of the cellulose through dehydration, decarboxylation, depolymerization followed by the formation of char residue^54,55^. The summary of the onset of degradation for this region and the remaining residue is shown in Table 4. The crosslinked foams showed an earlier onset of degradation compared to the uncrosslinked sample (Fig. 8a). This is possibly due to the presence of additional types of bonds (from the crosslinker; see Fig. 3) that break at a relatively lower temperature. Such a reduction in thermal stability as a result of the crosslinking was previously reported by our research group^56^. Among the tested samples (Fig. 8a,b), neat CNF and CNF:FLK foams had the lowest onset of degradation as opposed to the other two samples. The relatively lower onset in the CNF:CNC and CNF:CNC:FLK sample is possibly due to the sulphonate groups of the CNC which renders the CNC-containing foams thermally unstable^57^. One can easily observe a shoulder around 200–210 °C in the foams that contain CNC. On a related note, it was observed that when the CNC-containing foams were heated for crosslinking, the CNF:CNC sample had a slightly yellowish color, which is likely attributed to the same cause discussed above. Foams prepared from CNF:FLK and CNF:CNC:FLK had predictably the largest residue resulting from the presence of kaolin which starts to degrade at a relatively higher temperature (450–500 °C)^58^. DTGA curves show that, despite the late onset of degradation of neat CNF, the rate of degradation was higher than most of the other samples. The curves also show the relatively higher rate of degradation of CNC-containing samples at their early degradation.

**Table 4:** Summary of onset of degradation temperatures and remaining residue.

Sample name	Uncrosslinked		1 wt% crosslinker		5 wt% crosslinker	
	Onset of degradation (°C)	Residue (%)	Onset of degradation (°C)	Residue (%)	Onset of degradation (°C)	Residue (%)
Neat CNF	269	1.0	251	0.0	249	0.9
CNF:CNC	–	–	210	3.7	200	12.3
CNF:FLK	–	–	261	23.6	235	21.1
CNF:CNC:FLK	–	–	207	15.0	213	17.2

## Conclusions

Hybrid foams were prepared from different combinations of CNF, CNC, and kaolin-microfibrillated cellulose composite via freeze-drying and without additional functionalization. FTIR analysis verified the presence of ester linkages between epichlorohydrin (Polycup) and nanocellulose after heat curing of the foams. The fraction of cellulose nanofibrils, type of coexisting entity, and the crosslinker loading played major roles in the extent of shape recovery of the foams. The CNF:FLK foam (25 wt% kaolin) exhibited similar compressive properties to neat CNF foam, presumably resulting from the distinctive structure of the foam cells in this formulation. The presence of CNC at both the 50 wt% and 33 wt% led to a decrease in the surface area resulting from the formation of a non-porous and continuous network which filled the fibril-fibril and kaolin-fibrils voids. The preliminary comparative study of methylene blue removal from water demonstrated the potential of the prepared foams to be used in dye removal applications and highlighted the role of kaolin in enhancing the removal efficiency. Taken together, this study extends our knowledge of bio-based foams by understanding the relationship between the structure and properties of nanocellulose-kaolin clay foams. A natural progression of this work is to examine more closely the adsorption of methylene blue and potentially other contaminants.
